# A common variant of *CNTNAP2* is associated with sub-threshold autistic traits and intellectual disability

**DOI:** 10.1371/journal.pone.0260548

**Published:** 2021-12-13

**Authors:** Yuka Shiota, Tetsu Hirosawa, Yuko Yoshimura, Sanae Tanaka, Chiaki Hasegawa, Sumie Iwasaki, Kyung-min An, Daiki Soma, Masuhiko Sano, Shigeru Yokoyama, Mitsuru Kikuchi

**Affiliations:** 1 United Graduate School of Child Development, Osaka University, Kanazawa University, Hamamatsu University School of Medicine, Chiba University and University of Fukui, Kanazawa, Japan; 2 Research Center for Child Mental Development, Kanazawa University, Kanazawa, Japan; 3 Department of Psychiatry and Neurobiology, Graduate School of Medical Science, Kanazawa University, Kanazawa, Japan; 4 Institute of Human and Social Sciences, Kanazawa University, Kanazawa, Japan; Hamamatsu University School of Medicine, JAPAN

## Abstract

Sub-threshold autistic traits are common in the general population. Children with sub-threshold autistic traits have difficulties with social adaptation. Contactin-associated protein-like 2 (*CNTNAP2*) is associated with the development of Autism spectrum disorder (ASD) and the single-nucleotide polymorphism rs2710102 (G/A) of *CNTNAP2* is suggested to contribute to sub-threshold social impairments and intellectual disabilities. We recruited 67 children with Autistic disorder (AD) (49 boys, 18 girls, aged 38–98 months) and 57 typically developing (TD) children (34 boys, 23 girls, aged 53–90 months). We assessed the participants’ intelligence and social reciprocity using the Kaufman Assessment Battery for Children (K-ABC) and the Social Responsiveness Scale (SRS), respectively. Genomic DNA was extracted from the buccal mucosa and genotyped for rs2710102. A chi-square test revealed a significant association between genotype and group [χ^2^(2) = 6.56, p = 0.038]. When a co-dominant model was assumed, the results from linear regression models demonstrated that TD children with A-carriers (AA + AG) presented higher SRS T-scores [t(55) = 2.11, *p = 0*.*039*] and lower simultaneous processing scale scores of K-ABC [t(55) = -2.19, *p = 0*.*032*] than those with GG homozygotes. These associations were not significant in children with ASD. TD children with the rs2710102 A-allele may have more sub-threshold autistic traits than those with GG homozygotes, reflected in higher SRS scores and lower simultaneous processing scale scores. These results support the use of genetic evidence to detect sub-threshold autistic traits.

## Introduction

Autism spectrum disorder (ASD) is a neurodevelopmental disorder characterized by high heritability and deficits in social communication, restricted interests, and repetitive patterns of behavior [1]. Although the prevalence of ASD is estimated at only 1.85% [2], autistic social deficiency is common in the general population [3]. Given its continuous distribution, the boundary between typically developing (TD) children and children affected by ASD is somewhat ambiguous. In this context, children with less prominent autistic traits than those with fully diagnosed ASD are referred to as having sub-threshold autism traits [4]. Consequently, children with sub-threshold autistic traits tend to have difficulties in social adaptation, albeit they are not diagnosed with full-fledged ASD. The increasing evidence suggests that difficulties in social adaptation may result in an increased risk of comorbid mental health problems. For example, individuals with sub-threshold autistic traits have a higher prevalence of anxiety, depression, hyperactivity, problematic behavior, and more prone to substance abuse [5, 6]. Furthermore, sub-threshold autistic traits at age five predict later emotional symptoms and peer problems at age seven in the general population [7]. Overall, combined evidence from the literature suggest that early detection and appropriate support are necessary not only for children with ASD, but for those with sub-threshold autistic traits.

In line with the continuous distribution of autistic traits, almost all genetic risk factors for ASD can also be observed in the general population. Earlier studies have suggested associations between genetic risk factors and social impairments in children with ASD and the general population [8–10]. For example, Robinson et al. investigated several large ASD in consortia and the general population (total n > 38,000) and reported that multiple types of genetic risk for ASD influence a continuum of autistic traits, the severe tail of which can result in a diagnosis of ASD or other neuropsychiatric disorders [8]. From this perspective, although it is difficult to fully elucidate the genetic etiology of ASD considering its heterogeneous nature, utilizing evidence from genetic studies may be a promising approach to detect sub-threshold autistic traits.

Many genes, including common and rare variants, have been identified as the basis for ASD [11–13]. Among these, association, linkage, gene expression, and imaging studies suggest the role of both common and rare variants of contactin-associated protein-like 2 (*CNTNAP2*). Originally, a recessive nonsense mutation in *CNTNAP2* was observed to cause cortical dysplasia focal epilepsy syndrome, a syndromic form of ASD [14]. Several reports have linked this gene with an increased risk of autism [15, 16]. Among single nucleotide polymorphisms (SNPs) of *CNTNAP2*, rs2710102 (G/A, intron13) is one of the most frequently reported to be associated with autistic traits [17]. G-allele of rs2710102 has been associated with language impairments in individuals with and without ASD [18–20], but more importantly, having an A-allele of this SNP is associated with autistic traits in the general population [21–23], for example, having high AQ-social scores [21] and high social anxiety-related traits [22] in young adults (i.e., college students). Steer *et al*. reported that having the A-allele in children is associated with difficulties in inhibition in the context of social communication (i.e., social inhibition), albeit they did not directly investigate the relation between rs2710102 and autistic traits [23]. Although the association between rs2710102 and social and language impairment has been repeatedly demonstrated, Werling et al. meta-analyzed the association between rs2710102 and the diagnosis of ASD or high functioning ASD and reported that this association was non-significant [24]. Consequently, rs2710102 seems to confer sub-threshold autistic traits, the effect of which is sufficient to result in full-fledged ASD. This hypothesis encouraged us to utilize rs2710102 to detect individuals with sub-threshold autistic traits. Earlier detection and support have great merit in this context, however no previous study has directly focused on the association between rs2710102 and autistic traits during childhood. Furthermore, no studies have been conducted on the effect of rs2710102 on autistic symptoms in individuals with ASD. Therefore, the association between the A-allele and more prominent autistic symptoms remains unexplored in this population.

In addition to its association with autistic traits, biallelic defect in *CNTNAP2* has been reported to cause fully penetrant, severe forms of broad-spectrum neurodevelopmental disorders, such as cortical dysplasia and focal epilepsy syndrome [14, 25], and intellectual disability and epilepsy resembling Pitt-Hopkins syndrome [26]. Supporting the association between *CNTNAP2* and intellectual disability, Gregor et al. reported that even heterozygous variants or defects in *CNTNAP2* are associated with moderate to the severe intellectual disability with or without epilepsy [27]. Considering the association between *CNTNAP2* and intellectual disabilities, one might infer that rs2710102 itself is related to intelligence. Supporting this hypothesis, Uddin et al. recently conducted a case-control study followed by a meta-analysis and concluded that the presence of the G-allele of rs2710102 is associated with language impairment in children with and without ASD [28]. Similarly, the G-allele was associated with lower language skills in individuals with specific language impairment [12]. However, this putative association between rs2710102 and intelligence has not been directly examined. Notably, evidence suggests the complex relation among rs2710102, autistic traits, and language impairment. G-allele of rs2710102 is reported to be the risk factor for language impairment; however, A-allele is reported to be the risk factor for autistic traits.

In this study, we aimed to determine if and how rs2710102 is associated with sub-threshold autistic traits or intellectual disability in children with and without ASD. Considering the previous studies, we hypothesized that (i) carriers of the A-allele would have higher autistic traits compared to GG homozygotes in both populations and that (ii) carriers of the A-allele would also have higher intelligence compared to GG homozygotes in both populations.

## Materials and methods

### Participants

This study included 77 Japanese children with Autistic disorder (AD) and 57 TD children (91 boys and 43 girls, aged 38–98 months). Participants were recruited from Kanazawa University and affiliated hospitals. We confirmed that no TD child had a history of neurological, neuropsychiatric, or neurodevelopmental disorders. The diagnosis of AD was established according to the Diagnostic and Statistical Manual of Mental Disorders (4th edition) [29] using the Diagnostic Interview for Social and Communication Disorders [30] or the Autism Diagnostic Observation Schedule-Generic [31] or the Autism Diagnostic Observation Schedule 2 [32], details of which are given in the S1 and S2 Tables. We excluded participants with blindness, deafness, and other neuropsychiatric disorders, including epilepsy and ongoing medication. Intelligence is also an important factor in ASD, and high- and low-functioning autism are often distinguished based on intelligence quotient (IQ) scores [33]. “High functioning autism” is a term used for individuals with ASD who have an intelligence of 70 or above, especially indicating individuals without moderate to severe intellectual disability. ASDs with lower intelligence and those with higher intelligence are potentially different in terms of their cognitive profiles and genetic etiology. For example, Szatmari et al. (1998) reported that different genetic factors may account for the higher and lower functioning forms of autism [34]. Thus, we excluded 10 participants with an IQ <70. IQ was assessed using the mental processing composite scale of the Kaufman Assessment Battery for Children (K-ABC). Overall, we analyzed 67 children with AD (49 boys, 18 girls, aged 38–98 months) and 57 TD children (34 boys, 23 girls, aged 53–90 months). These are also part of an ongoing project (Bambi plan, http://bambiplan.w3.kanazawa-u.ac.jp/pdf/jusen_english.pdf). We are continually recruiting participants as part of a large epidemiological study, and some participants overlapped with those in our earlier study [35]. However, their results did not overlap. Additionally, the emphases of the earlier study differed from those of this study. The study was approved by the institutional review board of Kanazawa University Hospital and conducted per tenets of the Declaration of Helsinki [36]. Written informed consent was obtained from all participants’ guardians.

### Genotyping

Buccal mucosa cells were obtained from all participants by gently scraping the mucosa with a cotton swab. DNA was extracted from the cells, followed by whole genome amplification using the PicoPLEX WGA kit (Takara Bio, Mountain View, CA, USA). DNA concentrations were measured using a Qubit4 fluorometer (Thermo Fisher Scientific, Waltham, MA, USA). Genotyping of rs2710102 was performed by real-time PCR (ViiA 7, Applied Biosystems, Foster City, CA, USA) using the rhAmp SNP Genotyping system (assay name: CD.GT.TKKG0518.1: Integrated DNA Technologies, Coralville, IA, USA). The following RNA-DNA hybrid primers were used: 5′-TTGGTTAACATTTACTCTGAGACC**[T/C]**GAGAA-3′, and 5′-GCTGGAGTGAACCTGTTTGATTATT**G**CTGAT-3′ as locus-specific primers (https://sg.idtdna.com/pages/products/qpcr-and-pcr/genotyping/rhamp-snp-genotyping).

### Assessment of social reciprocity

The Social Responsiveness Scale (SRS) [37] was used to assess the participants’ social reciprocity: A 65-item rating scale that measures sociality and autistic mannerisms as a quantitative trait in TD children and children with ASD [38–40]. It measures subscales of social awareness, social cognition, social communication, social motivation, and autistic mannerisms to generate a single measure. We used gender-normed social responsiveness scale T scores (SRS-T) of each subscale [41]. Higher scores indicated greater difficulties in social reciprocity. The SRS has been one of the most frequently used quantitative measures of ASD symptoms, with very strong measurement properties in healthy volunteers and clinical cases of ASD [42]. In this context, this questionnaire is suitable for measuring sub-threshold autistic traits.

The participants’ parents filled out this questionnaire indicating how strongly they agreed with each question by checking one of the following: “never true,” “sometimes true,” “often true,” or “always true,” that were further scored as one, two, three, and four points, respectively [43]. Consequently, T-scores of SRS reflect the children’s social reciprocity in their natural social contexts [37]. It can describe moderate deficiencies in reciprocal social behavior that are “clinically significant” in ASD as well as in the general population [3].

### Assessment of intelligence

The Japanese version of the K-ABC [44], based on neuropsychological and information-processing theories, was used to assess the intelligence of all participants, with a distinction between problem-solving and knowledge of facts. The former was interpreted as intelligence, whereas the latter was defined as an achievement. Problem-solving abilities were measured using two mental processing scales [45], the sequential processing scale, which requires the child to solve problems in serial or temporal order, and the simultaneous processing scale, which requires the child to integrate multiple stimuli simultaneously to solve problems. The mental processing composite scale is a unification of the sequential and simultaneous processing scales, intended to measure total general intelligence. The achievement scale measures the degree of knowledge and skills that children acquire from the environment. These scores are provided as age-adjusted standardized scores normalized to a mean of 100 and a standard deviation of 15.

One of the goals of developing K-ABC was to separate intelligence from other factors, including achievement. Notably, although the achievement scale was reported to be well-correlated with language skills, the association of language skills with the two mental processing scales was reported to be weak. For example, Wiebe et al. reported that the correlation coefficient between the achievement scale and verbal IQ measured by Wechsler Intelligence Scale for Children-Revised was 0.77. In the same study, the correlation coefficient between sequential processing scale or simultaneous processing and verbal IQ were 0.54 and 0.63, respectively [46]. Since our objective was to examine the effect of rs2710102 on intelligence rather than on language skills, we focused on two mental processing scales in this study.

### Statistical analysis

Statistical analyses were performed using Stata software (ver. 16.1; Stata Corp., College Station, TX, USA). First, we checked the differences in age and scores in SRS-T and K-ABC between children with AD and TD using Student’s *t*-tests. Sex differences were tested using the chi-square test. Further, we employed a chi-square test to compare the group (AD or TD) and rs2710102 genotypes. Based on these results, we assumed a proper genetic model and evaluated the risk allele for AD. Based on the genetic model, we investigated the association between social reciprocity and the participants’ genotypic status after controlling for the disease group. In particular, we applied linear regression models to predict SRS total T-scores based on the participants’ group, genotype, and the interaction between group and genotype. If a significant interaction was observed or when appropriate, we investigated the association between genetic status and SRS-T score in each group. In addition to SRS total T-scores, as an exploratory analysis, we examined the correlation between each subscale of SRS and the genotype. In those models, we used each subscale of SRS (i.e., social awareness subscale, social cognitive subscale, social communication subscale, social motivation subscale, and autistic mannerisms sub-scale) as dependent variables instead of total SRS-T scores.

We also applied linear regression models to predict the K-ABC two mental processing scales (simultaneous processing scale and sequential processing scale) based on the participants’ group, genotype, and interaction with the group and adopted the same model for the achievement scale. A *p*-value <0.05 indicated statistical significance. If a significant interaction effect was observed or when appropriate, we applied post-hoc analysis to elucidate the association between the genotype and K-ABC scores in each group. In the post-hoc analyses, we predicted the sequential processing scale or simultaneous processing scale based on the participants’ group, genotype, and interaction with the group.

As an exploratory analysis, to control possible effects of SRS total T-scores on the scores in K-ABC or vice-versa, we analyzed the effect of genotype on SRS total T-scores controlling on the effect of intelligence. We further examined the effect of genotype on intelligence controlling for the effect of SRS total T-scores. Specifically, we applied linear regression models in each participant’s group (i.e., AD or TD) to predict (i) SRS total T-scores based on the genotype and simultaneous processing scale in K-ABC, (ii) SRS total T-scores based on the genotype and sequential processing scale in K-ABC, (iii) simultaneous processing scale in K-ABC based on the genotype and SRS total T-scores, and (iv) sequential processing scale in K-ABC based on the genotype and SRS total T-scores.

Before applying linear regression, we verified that our data met the assumptions for the regression analysis. Specifically, we used standard methods to verify linearity, normality, homogeneity of variance, model specifications, influence, and collinearity. However, the assumption of homogeneity was violated in some regression models. Therefore, we used heteroskedasticity-robust standard errors [47].

## Results

### Participants’ characteristics

The distribution of *CNTNAP2* rs2710102 (A/G) genotypes among the 124 sampled subjects were as follows: Twenty-eight participants were homozygous AA genotype, 67 participants carried the heterozygous AG, and 29 were homozygous GG. Tests for the Hardy-Weinberg equilibrium exhibited no deviations from the expected genotype distribution (*p* > *0*.*05*). The SRS total T-score was significantly higher in children with AD compared to that in TD children [*t*(120) = -12.23, *p < 0*.*001*]. The mental processing composite scale score was significantly lower in children with AD than in TD children [*t*(120) = 3.05, *p = 0*.*0027*]. This observation was consistent across sequential and simultaneous processing scales. The sequential processing scale and simultaneous processing scale scores were significantly lower in children with AD than in TD children [*t*(120) = 3.46, *p = 0*.*0007*; and *t*(120) = 2.40, *p = 0*.*0177*, respectively]. However, we observed no significant differences in achievement scale scores. The participant characteristics are presented in Table 1. There was no significant difference in age or sex between the groups.

**Table 1:** Participant characteristics.

	AD^1^	TD^2^	*χ*^*2*^ or *t*	*p*
	*N* = 67	*N* = 57		
Age in months	67.9 (11.6)	68.4 (9.2)	0.25	0.80
Sex (% Male)	59.0%	40.9%	2.53	0.11
SRS^3^ total T-score	71.4 (13.2)	47.3 (7.3)	-12.2	<0.001*
SRS sub-scores				
Social awareness	64.9 (10.0)	47.1 (8.7)	-10.5	<0.001*
Social cognition	71.8 (13.5)	49.4 (9.1)	-10.6	<0.001*
Social communication	68.5 (13.0)	46.5 (7.3)	-11.3	<0.001*
Social motivation	62.8 (14.0)	50.8 (7.9)	-5.8	<0.001*
Autistic mannerisms	73.6 (16.7)	45.7 (7.2)	-11.7	<0.001*
K-ABC^4^ scores				
Mental Processing composite	98.8 (16.4)	107.1 (13.0)	3.05	0.003*
K-ABC sub-scores				
Sequential Processing scale	95.8 (15.4)	105.4 (15.3)	3.46	<0.001*
Simultaneous Processing scale	100.1 (15.8)	106.2 (11.6)	2.4	0.02*
Achievement scale	97.1 (17.6)	102.9 (15.0)	1.95	0.05

Numbers are mean (standard deviation) or count.

* represents *p < 0*.*05*.

^1^AD, autistic disorder;

^2^TD, typically developing children;

^3^SRS, Social Responsiveness scale;

^4^K-ABC, Kaufman Assessment Battery for Children.

### Association between participant groups and genotypes

A chi-square test revealed a significant difference between the genotypes and groups (χ^2^(2) = 6.56, *p = 0*.*038*). Allele and genotype frequencies are presented in Table 2. The genetic model was determined based on these results. According to the selected genetic model [48], we transformed the three genotypes (AA, AG, and GG) into two variables. In particular, considering our results and the findings of previous studies [21], we assumed the co-dominant model: Carriers of the A-allele (AA + AG) versus G-allele homozygotes. This indicates that allele A increases autistic traits. Additionally, we employed a chi-square test to investigate the difference between carriers of the A-allele and the groups. However, this difference was not significant (χ^2^(2) = 1.29, *p = 0*.*256*).

**Table 2:** Allele and genotype frequencies.

Genotype	AD	TD	χ^2^	*p*
	*N* = 67 (%)	*N* = 57 (%)		
A/A	21 (31.3)	7 (12.2)	–	–
A/G	33 (49.2)	34 (59.6)	–	–
G/G	13 (19.4)	16 (28.0)	6.56	0.038*
Carriers of the A-allele	54 (80.5)	41 (71.9)	–	–
G-allele homozygotes	13 (19.4)	16 (28.0)	1.29	0.26

Numbers are counts (percentage).

* represents *p* < *0*.*05*.

### Association between genotypes and SRS total T-scores

**Fig 1:**
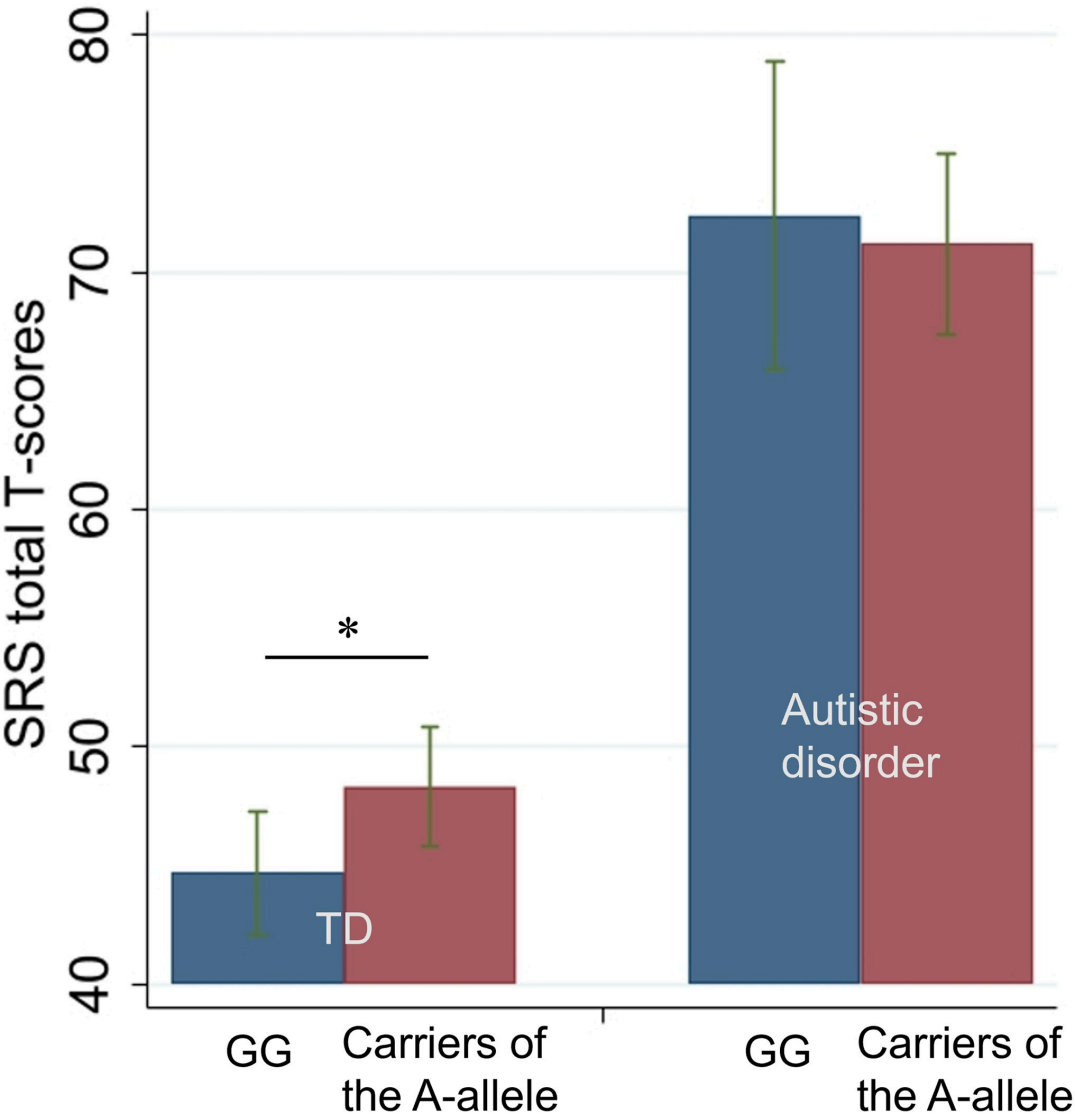
Bar charts present the SRS total T-scores between genotypes in children with autistic disorder (AD) and typically developing (TD) children (mean ± SD).

We applied linear regression models to predict the SRS total T-score based on the participants’ group (AD or TD), genotype (carriers of the A-allele or G-allele homozygotes), and interaction between group and genotype. Table 3 and Fig 1 summarize results of regression models. In this model, carriers of the A-allele and the group were significantly affected [*t*(120) = 2.11, *p* = *0*.*036*] and [*t*(120) = 8.82, *p* < *0*.*001*], respectively, details of which are given in the S1 Fig.

**Table 3:** Association between genotypes on SRS-T scores.

SRS total T scores	Coef.	Robust SE.	t	p	95% CI	*F*	*p*	*R* ^*2*^
Carrier of the A-allele	3.62	1.72	2.11	0.036*	0.22–7.03	65.03	<0.001*	0.56
Group (AD or TD)	27.70	3.13	8.82	<0.001*	21.48–33.91			
Group (AD or TD) × Carrier of the A-allele	-4.81	3.87	-1.24	0.22	-12.47–2.85			
Children with AD								
Carrier of the A-allele	-1.18	3.46	-0.34	0.73	-8.1–5.74	0.12		0.00
TD children								
Carrier of the A-allele	3.62	1.72	2.11	0.039*	0.17–7.08	4.44		0.05

AD, autistic disorder; SRS, Social Responsiveness Scale; TD, typically developing children.

Additionally, the *F* statistic was significant [*F*(3, 120) = 65.63, *p* < *0*.*001*]. However, the interaction was not significant [*t*(120) = -1.24, *p* = *0*.*216*]. For this particular model, the assumption of normality was violated. According to the histogram, this was caused by different patterns of association in AD or TD. Hence, we applied linear regression to predict the SRS total T-score based on genotypes in each group. As a result, a significant main effect of genotype was observed for TD [*t*(55) = 2.11, *p* = *0*.*039*]. However, this association was not significant in children with AD.

As an exploratory analysis, to further investigate the association between the genotype and social reciprocity, we examined the correlation between each subscale of SRS and the genotype. Aside from the main effect of the participants’ group, we observed a significant effect only for the model predicting social awareness subscale. Specifically, we observed a main effect of the genotype to be significant [t(120) = 1.99, *p = 0*.*049*] for the model, indicating that carriers of the A-allele had a higher social awareness subscale of SRS. Post-hoc analysis demonstrated that this effect was larger in the TD group, although it did not reach statistical significance [t(55) = 1.98, *p = 0*.*52*]. The results are presented in S3 Table.

### Association between genotypes on K-ABC scores

For the model predicting the mental processing composite scale based on the participants’ group (AD or TD), genotype (carriers of the A-allele or G-allele homozygotes), and interaction with the group, only the main effect of the group was observed to be significant [*t*(120) = -2.74, *p* = *0*.*007*]. No other factors were observed to be statistically significant. For the model predicting the simultaneous processing scale, the main effect of genotype was observed to be significant [*t*(120) = -2.19, *p* = *0*.*030*], and the group [*t*(120) = -3.43, *p* < *0*.*001*] was also observed to be significant. The interaction effect was also significant [*t*(120) = 2.55, *p = 0*.*011*]. Hence, we applied a post-hoc analysis to predict the simultaneous processing scale based on the genotypes in each group, and a significant main effect of genotype was observed only for TD [*t*(55) = -2.19, *p* = *0*.*033*], scatter plot of which are provided in the S2 Fig. Table 4 summarizes the results of the models predicting the simultaneous processing scale. For the model predicting the sequential processing scale, no factor was observed to be significant.

**Table 4:** Association between genotypes on simultaneous processing scale of K-ABC.

Sequential processing scale	Coef.	Robust SE.	*t*	*p*	95% CI		*F*	*p*	R^2^
Group (AD or TD)	-8.23	4.70	-1.75	0.08	-17.53	1.07	4.09	0.01	0.09
Carrier of the A-allele	2	4.23	0.47	0.64	-6.37	10.37			
Group (AD or TD) × Carrier of the A-allele	-1.95	5.78	-0.34	0.74	-13.40	9.49			
Simultaneous processing scale	Coef.	Robust SE.	*T*	*p*	95% CI		*F*	*P*	R^2^
Group (ASD or TD)	-17.48	5.09	-3.43	0.001*	-27.56	-7.40	4.84	0.003*	0.09
Carrier of the A-allele	-6.93	3.16	-2.19	0.03*	-13.19	-0.68			
Group (AD or TD) × Carrier of the A-allele	14.85	5.81	2.55	0.012*	3.34	26.36			
Children with AD	Coef.	Robust SE.	*T*	*p*	95% CI		*F*	*P*	R^2^
Carrier of the A-allele	7.92	4.87	1.62	0.11	-1.82	17.65	2.64		0.04
TD children									
Carrier of the A-allele	-6.93	3.16	-2.19	0.033*	-13.27	-0.59	4.80		0.07

AD, autistic disorder; K-ABC, Kaufman Assessment Battery for Children; TD, typically developing children.

#### Association between genotypes and SRS total T-scores controlling for the effect of K-ABC scores, and the association between genotypes and K-ABC scores controlling for the effect of SRS total T-scores

Results were slightly similar to those presented above. In TD children, carriers of the A-allele had significantly higher SRS total T-scores after controlling for the effect of sequential processing scale [t(54) = 2.10, *p = 0*.*04*], however statistical significance was not seen while controlling for the effect of simultaneous processing scale [t(54) = 1.78, *p = 0*.*08*]. For the models predicting simultaneous processing scores, carriers of the A-allele had significantly lower simultaneous processing scores after controlling for the effect of SRS total T-scores [t(64) = -2.17, *p = 0*.*03*]. In children with AD, no significant effect was observed. The results are presented in S4 Table.

## Discussions

To our knowledge, this is the first study to reveal a significant association between common variants of the autism-related gene, rs2710102 in *CNTNAP2* and autistic traits and intelligence in TD Japanese children. Particularly, we observed that TD children carrying the A-allele presented significantly higher SRS total T-scores compared to G-allele homozygotes. We also observed that TD children carrying the A-allele had significantly lower simultaneous processing scores compared to G-allele homozygotes. However, these associations were not significant for children with AD.

Our results indicated that carriers of the A-allele have high SRS total T-scores in TD children; however, this relation was non-significant in children with AD. This result was consistent with the previous findings in young adults (i.e., college students), where carriers of the A-allele are associated with social difficulties, including high AQ-social scores [21] and social anxiety-related traits [22]. In this context, our results confirmed the association between the presence of A-allele and autistic traits, extending it to the younger population (i.e., 38–98 months). In a similar age range, Steer et al. examined 13,138 children aged 6 months to 9 years old and reported that the presence of A-allele is associated with difficulties in social inhibition [23], however they did not directly investigate the relation between rs2710102 and autistic traits. Therefore, this study was the first to demonstrate that TD children carrying the A-allele of rs2710102 already have subclinical autistic traits as determined by the SRS, since early childhood. Further studies are thus needed to investigate how such subtle social difficulties affect their functional outcomes in adulthood. From a clinical perspective, this population might benefit from interventions that focus on boosting social-emotional, cognitive, and language abilities (e.g., early start Denver model). Future clinical trials that utilize rs2710102 to detect children with subthreshold autistic traits will be beneficial to confirm the efficacy of such interventions.

The association between rs2710102 and autistic traits, however, was not significant in children with AD. This discrepancy can be explained by the genetic characteristics of AD. AD is with a wide variety of mutations and consequently, autistic symptoms are affected by many genes. In this context, autistic traits might have already been “saturated” in children with full-fledged AD, and thus, one SNP in *CNTNAP2* may not be sufficient to significantly alter social reciprocity in this population. Alternatively, the effect of this SNP on autistic traits can be buffered by other autism genes and their molecular pathways. In this case, rs2710102 may exert its effect on interactions with other genes, some of which (possibly autism genes) may mitigate their effects on autistic traits, while others may exacerbate them. Further studies focusing on the relation between rs2710102 and autistic traits in other developmental disorders will be beneficial to clarify whether this polymorphism specifically affects TD children.

In addition to social reciprocity, unlike our hypothesis, we observed significantly lower simultaneous processing scores in carriers of the A-allele in TD children, whereas the effect was non-significant in children with AD. This result was consistent with the previous findings revealing that balletic defects [26] or heterozygous variant or defects in *CNTNAP2* are associated with moderate to severe intellectual disability [27]. Additionally, our results provided another evidence supporting the role of *CNTNAP2* in intelligence, by presenting that the association may even be caused by a single SNP (i.e., rs2710102). The risk allele was different from that for language impairment. In contrast to our results, the G-allele has been observed to be associated with language impairment in children with AD [28], as well as specific language impairment [19], and in the general population [17]. Our results thus extend those previous findings in two meaningful ways: (i) The effect of this SNP in rs2710102 on brain function is not only limited to language skills in TD children, but also extends to intelligence and autistic traits. In children with AD, however, its effect on intelligence and autistic traits would not be as strong as that in TD children. (ii) The effect of this SNP on brain function is not straightforward. Carriers of the A-allele of rs2710102 may have better language skills, albeit they also have lower intelligence and more prominent autistic traits. Overall, the complex structure of the relation between rs2710102, intelligence, autistic traits and language skills is remarkable. The effect of rs2710102 on brain function and risk allele seem to be different depending on the presence of AD and the domain of the function (i.e., A-allele for intelligence and autistic traits, G-allele for language skills), respectively. These results support the hypothesis that rs2710102 exerts its effect on complex interactions with other genes, such that autism genes may not only affect the autistic traits, but also intelligence.

The sequential and simultaneous cognitive processes have been associated with specific anatomical regions of the brain [49–51]. In general, the left hemisphere is responsible for processing language and performing sequential processing of information [52]. Meanwhile, the right hemisphere is considered specialized for simultaneous processing [52]. Consequently, our findings could be attributable to right hemispheric dysfunction in carriers of the A-allele. Although the right hemisphere has been considered the nonverbal side, it helps language processes in the left hemisphere and plays a role in cognitive-communication [53]. In particular, the right hemisphere appears to be critical for pragmatic communication [54]. Pragmatic communication refers to the ability to use language in context. For example, it includes the ability of verbal and non-verbal communication and the ability to use context cues for understanding verbal and non-verbal communication [55]. Notably, impairment of these abilities is an essential feature of AD. Indeed, high-functioning autism has been linked with a communication disorder known as a semantic-pragmatic disorder, speculated as a dysfunction of the right hemisphere [56–58]. From this perspective, the observed subclinical autistic traits in TD children carrying the A-allele could also be attributable to right hemispheric dysfunction, which results in impaired pragmatic communication.

The association between *CNTNAP2* and the right hemisphere further supports this view. For example, G-allele homozygotes of rs2710102 reportedly have significantly increased activation of the right inferior frontal gyrus during a language task in healthy adults compared to carriers of the A-allele [20]. Carriers of the G-allele demonstrate greater right frontal connectivity compared to non-G-carriers [59]. Furthermore, evidence suggests that even a single SNP in *CNTNAP2* may be sufficient to cause a significant change in right hemisphere function. For example, Riva et al. reported an association of the rs2710102 G/G genotype with lower P3 amplitude in the right hemisphere during rapid auditory processing, which in turn predicted later poor expressive vocabulary [18]. Koeda et al. reported that A/T heterozygotes in rs7794745, another SNP of *CNTNAP2*, presented a reduction in the right frontal activity of language processing compared to A/A homozygotes in healthy adults [60]. Combining all the evidence, based on the presumable association between rs2710102 of *CNTNAP2* and the right hemisphere, it can be hypothesized that in TD individuals, *CNTNAP2* polymorphism affects functions of the right hemisphere. Therefore, carriers of the A-allele of rs2710102 would affect the functions of the right hemisphere, causing lower simultaneous processing scores.

### Limitations

This study has several limitations. First, the sample size was too small to investigate behavioral characteristics. Larger samples are thus needed to clarify characteristics of autistic traits in TD children. Second, we did not use multiple tests in the current study. Most genetic studies with relatively few polymorphisms are not sufficiently powered to derive definitive conclusions when adjusting the results for multiple testing [61]. Further, adjusting for multiple tests may increase the chance of type II errors owing to the conservative nature of the Bonferroni adjustment [62]. Third, we need to confirm the association between these results and neural substrates. The ongoing study may assist in providing more evidence on genetic-behavior-brain interactions using neuroimaging methods. Fourth, we could not prove a cause-and-effect association between the genetic changes and behavioral characteristics. Further studies are thus required to elucidate the underlying biological mechanisms. Moreover, all our participants were children, and we did not include any adolescents or adults. These results should not be generalized unless validated in older individuals. Fifth, although the K-ABC second edition (K-ABC II, [63]) was released in 2004, we used the original version of K-ABC [64]. Similarly, we used the original version of SRS rather than SRS-2 [41], which is available from 2017. This was because the Japanese version of neither the K-ABC second edition nor SRS second edition had been verified at the time we started this study. However, in any case, this did not align with the current best practice standards. Sixth, all the TD children were native Japanese with no previous or existing developmental, learning, or behavioral problems according to information obtained from questionnaires completed by their parents. Consequently, it might be possible that some children we classified as TD had AD. This should be especially considered for those who had higher SRS total T-scores.

## Conclusions

Our findings suggest that genetic variation in rs2710102 of *CNTNAP2* is associated with sub-threshold autistic traits and intelligence in TD children. One issue of practical interest is the possible use of evidence from genetic studies to detect sub-threshold autistic traits. This application is attractive in clinical practice, however it still needs to overcome reasonable limitations (e.g., determining who requires appropriate support or the extent to which individuals are in need). Additional studies need to be conducted in this respect.

## Supporting information

S1 Fig Scatter plots of the distribution of the variance in SRS total T-scores. Scatter plots present the SRS total T-scores for carriers of the A-allele and GG genotype among children with autistic disorder (AD) and typically developing (TD) children. Some children in the AD group had lower (<60) SRS total T-scores, albeit their diagnosis of AD was confirmed using ADOS or DISCO, both of which are the golden standards for diagnosing ASD.
(TIFF)

S2 Fig Scatter plots of the distribution in the simultaneous processing scale. Scatter plots present the simultaneous processing scale for carriers of the A-allele and GG genotype among children with autistic disorder (AD) and typically developing (TD) children.
(TIFF)

S1 Table Scores and sub-scores of ADOS in children with autistic disorder. (DOCX)

S2 Table Scores and sub-scores of ADOS-2 in children with autistic disorder. (DOCX)

S3 Table Association between genotypes and sub-scales of SRS. (DOCX)

S4 Table Association between genotypes and SRS total T-scores controlling for the effect of K-ABC scores, and association between genotypes and K-ABC scores controlling for the effect of SRS total T-scores. (DOCX)

S1 Raw data (XLSX)
