# Supplementary figures and images for: A common variant of CNTNAP2 is associated with sub-threshold autistic traits and intellectual disability
Source: PLoS One. 2021 Dec 13;16(12):e0260548. doi: 10.1371/journal.pone.0260548 (PMC8668106; doi:10.1371/journal.pone.0260548)

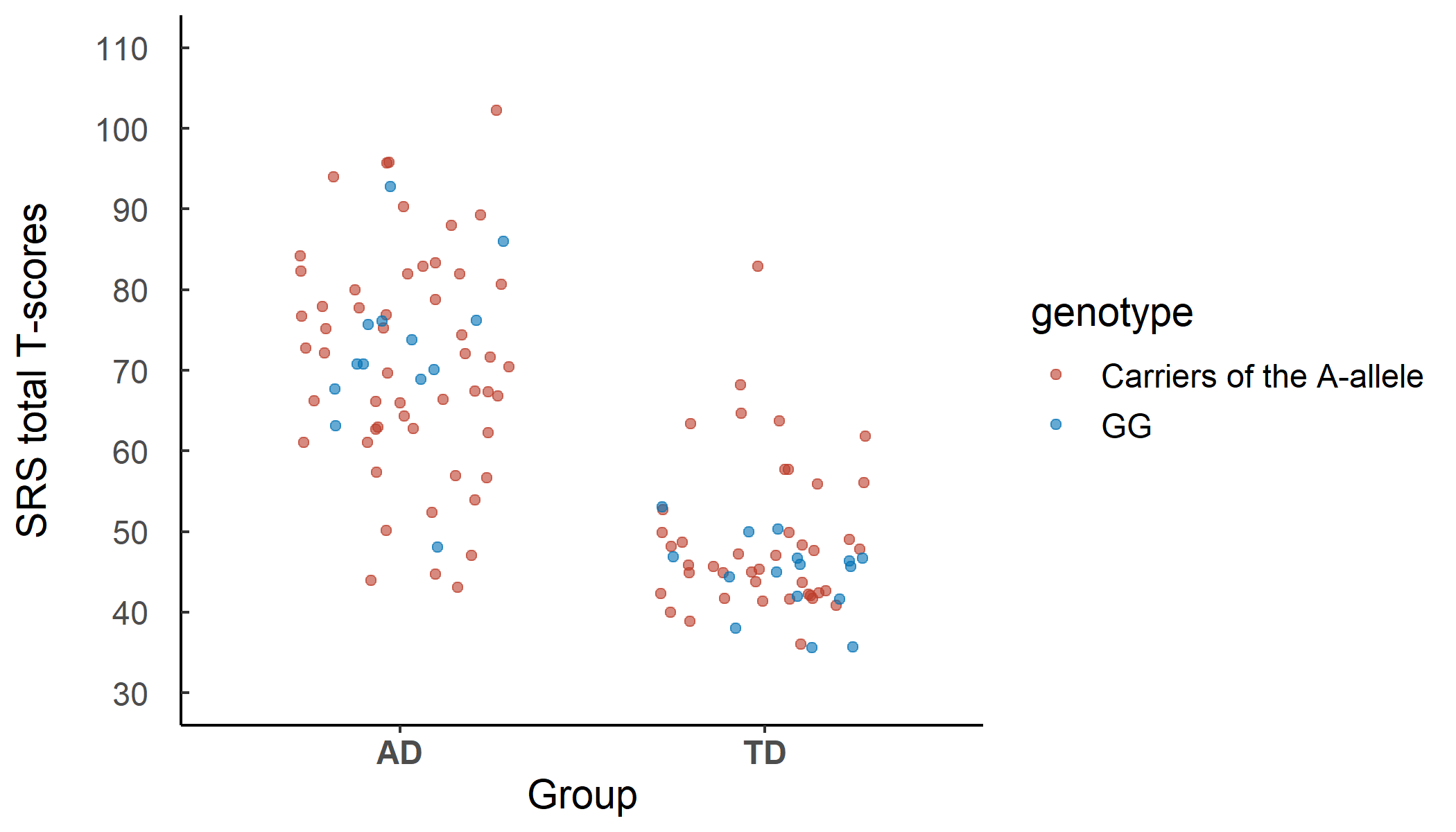

Supplement: S1 Fig — Scatter plots present the SRS total T-scores for carriers of the A-allele and GG genotype among children with autistic disorder (AD) and typically developing (TD) children. Some children in the AD group had lower (<60) SRS total T-scores, albeit their diagnosis of AD was confirmed using ADOS or DISCO, both of which are the golden standards for diagnosing ASD. (TIFF) [file pone.0260548.s001.tiff]

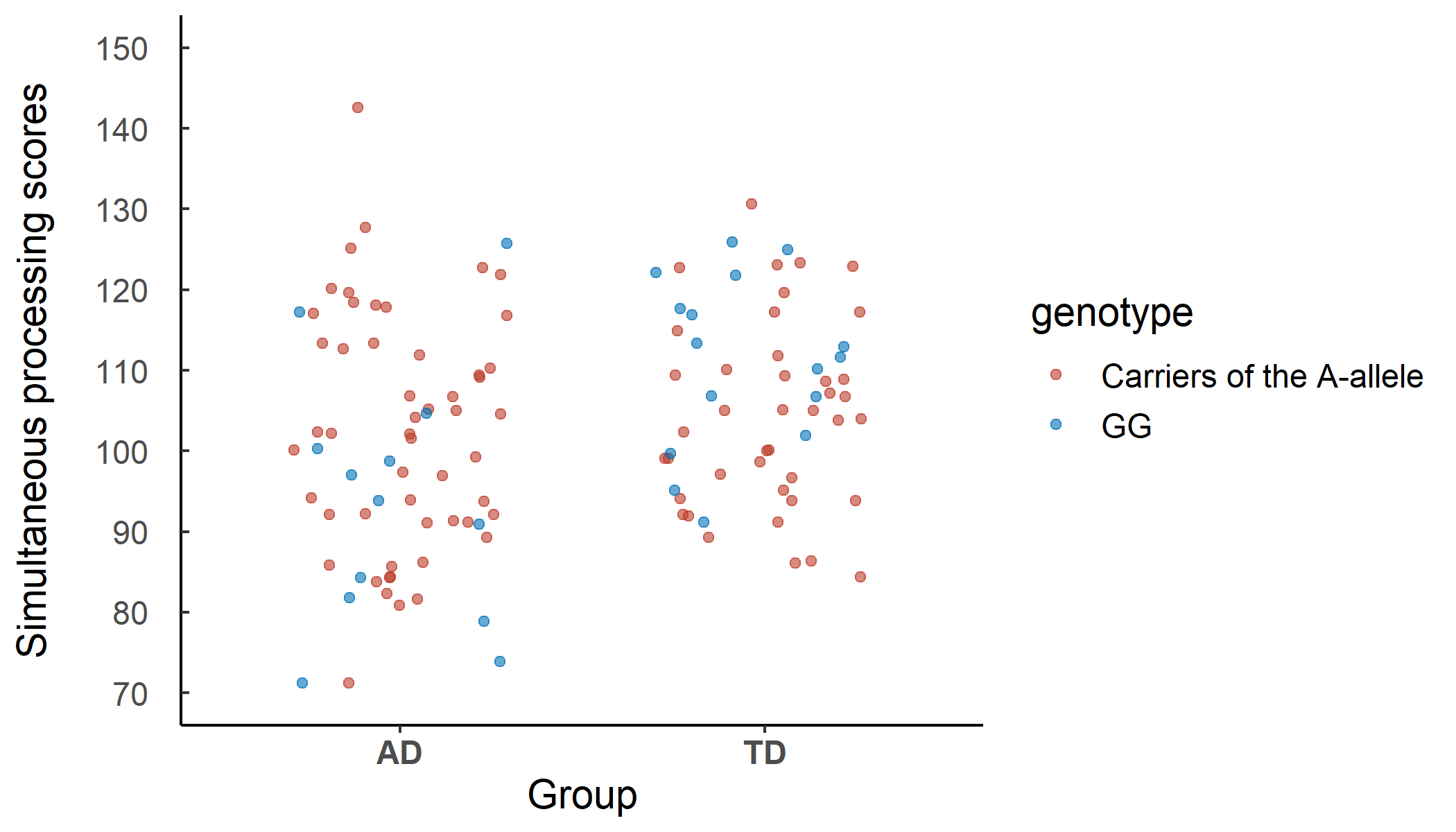

Supplement: S2 Fig — Scatter plots present the simultaneous processing scale for carriers of the A-allele and GG genotype among children with autistic disorder (AD) and typically developing (TD) children. (TIFF) [file pone.0260548.s002.tiff]
