# Supplementary material for: A common variant of CNTNAP2 is associated with sub-threshold autistic traits and intellectual disability
Source: PLoS One. 2021 Dec 13;16(12):e0260548. doi: 10.1371/journal.pone.0260548 (PMC8668106; doi:10.1371/journal.pone.0260548)
Supplement: S1 Table — (DOCX) [file pone.0260548.s003.docx]

**Supplementary Table 1. Scores and sub-scores of ADOS in children with autistic disorder**

|  | Module1  ASD *N*= 3 | Module2 ASD *N*= 30 | Module3 ASD *N*= 1 |
| --- | --- | --- | --- |
| ADOS scores |  |  |  |
| Communication | 4.0 (0.82) | 4.00 (1.58) | 6 (0) |
| Interaction | 6.67 (3.09) | 8.00 (1.55) | 13 (0) |
| total  (Communication + Interaction) | 10.67 (3.30) | 12.00 (2.76) | 19 (0) |
| Imagination / Creativity | 1.67 (1.70) | 1.00 (0.65) | 3 (0) |
| Stereotypic behavior /  Restricted Interest | 1.33 (0.47) | 1.00 (0.88) | 1 (0) |
|  |  |  |  |
| Numbers are mean (standard deviation). | | | |
