# Supplementary material for: A common variant of CNTNAP2 is associated with sub-threshold autistic traits and intellectual disability
Source: PLoS One. 2021 Dec 13;16(12):e0260548. doi: 10.1371/journal.pone.0260548 (PMC8668106; doi:10.1371/journal.pone.0260548)
Supplement: S2 Table — (DOCX) [file pone.0260548.s004.docx]

**Supplementary Table 2. Scores and sub-scores of ADOS-2 in children with autistic disorder**

|  | Module2 ASD *N*= 17 | Module3 ASD *N*= 2 |
| --- | --- | --- |
| ADOS-2 scores |  |  |
| SA | 7.71 (2.63) | 8.00 (0) |
| RRB | 2.24 (1.52) | 1.50 (1.50) |
| Total | 9.94 (3.28) | 9.50 (1.50) |
| CS | 5.41 (1.50) | 6.00 (1) |
|  |  |  |
| Numbers are mean (standard deviation). | | |
