# Supplementary material for: A common variant of CNTNAP2 is associated with sub-threshold autistic traits and intellectual disability
Source: PLoS One. 2021 Dec 13;16(12):e0260548. doi: 10.1371/journal.pone.0260548 (PMC8668106; doi:10.1371/journal.pone.0260548)
Supplement: S3 Table — (DOCX) [file pone.0260548.s005.docx]

**Supplementary Table 3. Association between genotypes and sub-scales of SRS**

|  | Coef. | Robust SE. | t | p | 95% CI | | | F | p | R^2^ |
| --- | --- | --- | --- | --- | --- | --- | --- | --- | --- | --- |
| **Autistic Mannerisms** |  |  |  |  |  |  |  |  |  |  |
| Group  (AD or TD) | 31.22 | 4.14 | 7.55 | <0.001 | 23.03 | - | 39.41 | 61.71 | <0.001 | 0.53 |
| Carrier of the | 2.40 | 1.52 | 1.58 | 0.117 | -0.61 | - | 5.42 |  |  |  |
| A-allele |  |  |  |  |  |  |  |  |  |  |
| Group  (AD or TD) | -4.34 | 4.93 | -0.88 | 0.38 | -14.1 | - | 5.43 |  |  |  |
| × |  |  |  |  |  |  |  |  |  |  |
| Carrier of the |  |  |  |  |  |  |  |  |  |  |
| A-allele |  |  |  |  |  |  |  |  |  |  |
| Children with AD |  |  |  |  |  |  |  |  |  |  |
| Carrier of the  A-allele | -1.93 | 4.68 | -0.41 | 0.68 | -11.28 | - | 7.42 | 0.17 |  | 0.00 |
| TD children |  |  |  |  |  |  |  |  |  |  |
| Carrier of the  A-allele | 2.40 | 1.53 | 1.58 | 0.12 | -0.65 | - | 5.46 | 2.48 |  | 0.02 |
|  |  |  |  |  |  |  |  |  |  |  |
| **Social Motivation** |  |  |  |  |  |  |  |  |  |  |
| Group  (AD or TD) | 16.15 | 3.68 | 4.39 | <0.001 | 8.86 | - | 23.44 | 14.59 | <0.001 | 0.22 |
| Carrier of the  A-allele | 3.42 | 2.03 | 1.68 | 0.095 | -0.61 | - | 7.45 |  |  |  |
| Group  (AD or TD) | -5.49 | 4.37 | -1.26 | 0.21 | -14.15 | - | 3.17 |  |  |  |
| × |  |  |  |  |  |  |  |  |  |  |
| Carrier of the  A-allele |  |  |  |  |  |  |  |  |  |  |
| Children with AD |  |  |  |  |  |  |  |  |  |  |
| Carrier of the  A-allele | -2.07 | 3.87 | -0.54 | 0.59 | -9.80 | - | 5.65 | 0.29 |  | 0.00 |
| TD children |  |  |  |  |  |  |  |  |  |  |
| Carrier of the  A-allele | 3.42 | 2.04 | 1.68 | 0.099 | -0.66 | - | 7.50 | 2.82 |  | 0.04 |
|  |  |  |  |  |  |  |  |  |  |  |
| **Social Communication** |  |  |  |  |  |  |  |  |  |  |
| Group  (AD or TD) | 24.01 | 3.70 | 6.48 | <0.001 | 16.67 | - | 31.34 | 50.16 | <0.001 | 0.52 |
| Carrier of the  A-allele | 2.53 | 1.81 | 1.40 | 0.165 | -1.06 | - | 6.12 |  |  |  |
| Group  (AD or TD) | -2.78 | 4.30 | -0.65 | 0.519 | -11.30 | - | 5.74 |  |  |  |
| × |  |  |  |  |  |  |  |  |  |  |
| Carrier of the  A-allele |  |  |  |  |  |  |  |  |  |  |
| Children with AD |  |  |  |  |  |  |  |  |  |  |
| Carrier of the  A-allele | -0.25 | 3.90 | -0.06 | 0.949 | -8.03 | - | 7.53 | 0.00 |  | 0 |
| TD children |  |  |  |  |  |  |  |  |  |  |
| Carrier of the  A-allele | 2.53 | 1.82 | 1.39 | 0.169 | -1.11 | - | 6.17 | 1.94 |  | 0.03 |
|  |  |  |  |  |  |  |  |  |  |  |
| **Social Cognition** |  |  |  |  |  |  |  |  |  |  |
| Group  (AD or TD) | 27.22 | 3.75 | 7.26 | <0.001 | 19.80 | - | 34.64 | 47.07 | <0.001 | 0.49 |
| Carrier of the  A-allele | 3.36 | 2.18 | 1.54 | 0.127 | -0.96 | - | 7.67 |  |  |  |
| Group  (AD or TD) | -6.29 | 4.48 | -1.41 | 0.163 | -15.15 | - | 2.57 |  |  |  |
| × |  |  |  |  |  |  |  |  |  |  |
| Carrier of the  A-allele |  |  |  |  |  |  |  |  |  |  |
| Children with AD |  |  |  |  |  |  |  |  |  |  |
| Carrier of the  A-allele | -2.93 | 3.90 | -0.75 | 0.455 | -10.73 | - | 4.86 | 0.56 |  | 0.01 |
| TD children |  |  |  |  |  |  |  |  |  |  |
| Carrier of the  A-allele | 3.36 | 2.18 | 1.54 | 0.13 | -1.02 | - | 7.73 | 2.36 |  | 0.03 |
|  |  |  |  |  |  |  |  |  |  |  |
| **Social Awareness** |  |  |  |  |  |  |  |  |  |  |
| Group  (AD or TD) | 18.87 | 2.59 | 7.28 | <0.001 | 13.73 | - | 24.0 | 43.07 | <0.001 | 0.49 |
| Carrier of the  A-allele | 4.59 | 2.31 | 1.99 | 0.049* | 0.02 | - | 9.17 |  |  |  |
| Group  (AD or TD) | -1.71 | 3.28 | -0.52 | 0.604 | -8.20 | - | 4.79 |  |  |  |
| × |  |  |  |  |  |  |  |  |  |  |
| Carrier of the  A-allele |  |  |  |  |  |  |  |  |  |  |
| Children with AD |  |  |  |  |  |  |  |  |  |  |
| Carrier of the  A-allele | 2.89 | 2.33 | 1.24 | 0.219 | -1.76 | - | 7.53 | 1.54 |  | 0.01 |
| TD children |  |  |  |  |  |  |  |  |  |  |
| Carrier of the  A-allele | 4.59 | 2.32 | 1.98 | 0.052 | -0.05 | - | 9.23 | 3.93 |  | 0.06 |

AD, autistic disorder; SRS, Social Responsiveness Scale; TD, typically developing children
