# Supplementary material for: A common variant of CNTNAP2 is associated with sub-threshold autistic traits and intellectual disability
Source: PLoS One. 2021 Dec 13;16(12):e0260548. doi: 10.1371/journal.pone.0260548 (PMC8668106; doi:10.1371/journal.pone.0260548)
Supplement: S4 Table — (DOCX) [file pone.0260548.s006.docx]

**Supplementary Table 4. Association between genotypes and SRS total T-scores controlling for the effect of K-ABC scores, and association between genotypes and K-ABC scores controlling for the effect of SRS total T-scores**

|  | Coeff | Robust SE | t | p | 95 % CI | | | F | p | R^2^ |
| --- | --- | --- | --- | --- | --- | --- | --- | --- | --- | --- |
| **Sequential Processing Scale** |  |  |  |  |  |  |  |  |  |  |
| Children with AD |  |  |  |  |  |  |  |  |  |  |
| SRS Total  T-scores | -0.02 | 0.16 | -0.12 | 0.907 | -0.34 | - | 0.30 | 0.01 | 0.99 | 0 |
| Carrier of the  A-allele | 0.02 | 3.94 | 0.01 | 0.995 | -7.84 | - | 7.88 |  |  |  |
| TD children |  |  |  |  |  |  |  |  |  |  |
| SRS Total  T-scores | 0.1 | 0.24 | 0.42 | 0.676 | -0.38 | - | 0.58 | 0.20 | 0.82 | 0.01 |
| Carrier of the  A-allele | 1.64 | 4.35 | 0.38 | 0.708 | -7.08 | - | 10.35 |  |  |  |
|  |  |  |  |  |  |  |  |  |  |  |
| **Simultaneous Processing Scale** |  |  |  |  |  |  |  |  |  |  |
| Children with AD |  |  |  |  |  |  |  |  |  |  |
| SRS Total  T-scores | -0.17 | 0.15 | -1.11 | 0.27 | -0.46 | - | 0.13 | 1.78 | 0.18 | 0.06 |
| Carrier of the  A-allele | 7.72 | 4.98 | 1.55 | 0.13 | -2.23 | - | 17.67 |  |  |  |
| TD children |  |  |  |  |  |  |  |  |  |  |
| SRS Total  T-scores | -0.04 | 0.26 | -0.15 | 0.878 | -0.55 | - | 0.47 | 2.41 | 0.1 | 0.07 |
| Carrier of the  A-allele | -6.79 | 3.12 | -2.17 | 0.034* | -13.05 | - | -0.53 |  |  |  |
|  |  |  |  |  |  |  |  |  |  |  |
| **SRS Total**  **T-scores** |  |  |  |  |  |  |  |  |  |  |
| Children with AD |  |  |  |  |  |  |  |  |  |  |
| Sequential Processing Scale | -0.01 | 0.12 | -0.12 | 0.908 | -0.26 | - | 0.23 | 0.06 | 0.94 | 0.00 |
| Carrier of the  A-allele | -1.18 | 3.49 | -0.34 | 0.736 | -8.16 | - | 5.79 |  |  |  |
| TD children |  |  |  |  |  |  |  |  |  |  |
| Sequential Processing Scale | 0.02 | 0.05 | 0.41 | 0.684 | -0.08 | - | 0.13 | 2.21 | 0.12 | 0.05 |
| Carrier of the  A-allele | 3.59 | 1.71 | 2.10 | 0.041* | 0.16 | - | 7.02 |  |  |  |
|  |  |  |  |  |  |  |  |  |  |  |
| **SRS Total**  **T-scores** |  |  |  |  |  |  |  |  |  |  |
| Children with AD |  |  |  |  |  |  |  |  |  |  |
| Simultaneous Processing Scale | -0.12 | 0.11 | -1.08 | 0.282 | -0.34 | - | 0.10 | 0.728 | 0.487 | 0.02 |
| Carrier of the  A-allele | -0.23 | 3.84 | -0.06 | 0.952 | -7.90 | - | 7.43 |  |  |  |
| TD children |  |  |  |  |  |  |  |  |  |  |
| Simultaneous Processing Scale | -0.02 | 0.10 | -0.15 | 0.878 | -0.23 | - | 0.19 | 2.28 | 0.112 | 0.05 |
| Carrier of the  A-allele | 3.52 | 1.97 | 1.78 | 0.08 | -0.44 | - | 7.48 |  |  |  |

AD, autistic disorder; SRS, Social Responsiveness Scale; TD, typically developing children
